# Creation of an ex-vivo bovine kidney flow model for testing embolic agents: work in progress

**DOI:** 10.1186/s42155-021-00210-0

**Published:** 2021-02-03

**Authors:** Luis Garza, Ryan Bitar, Barrett O’Donnell, Matthew Parker, Carlos Ortiz, Charles Hyman, John Walker, Ho-Young Song, Jorge Lopera

**Affiliations:** 1 Long School of Medicine, University of Texas Health Science Center, 7703 Floyd Curl Drive San Antonio, San Antonio, TX 78229 USA; 2 Department of Radiology, University of Texas Health Science Center, San Antonio, TX USA; 3 Department of Diagnostic Radiology, Asan Medical Center, University of Ulsan College of Medicine, Seoul, Republic of Korea

**Keywords:** Flow model, Perfusion, Bovine kidney, Embolization, Fluoroscopy

## Abstract

**Objectives:**

To develop an ex- vivo perfusion flow model using a bovine kidney for future testing of embolic agents in an inexpensive and easy way.

**Methods:**

Six bovine adult kidneys were used for this study. Kidneys were cannulated and perfused via a roller pump. Three embolic agents, coils, Gelfoam, and a glue mixture of Histoacryl + Lipiodol, were deployed by targeting three secondary segmental arteries per kidney via a 5Fr catheter under fluoroscopic guidance. Cannulation time, success rate of segmental artery selection and embolic agent deployment, total operational time, and fluoroscopy dose were recorded.

**Results:**

Average kidney weight was 0.752 +/− 0.094 kg. All six bovine kidneys were successfully cannulated in 21.6 min +/− 3.0 min. Deployment of coils and glue was achieved in every case (12/12); however, Gelfoam injection was not successful in one instance (5/6, 83%). Coil deployment demonstrated no embolic effect while Gelfoam and glue injections demonstrated decreased distal contrast filling post-embolization. Mean dose area product was 12.9 ± 1.8 Gy·cm2, fluoroscopy time was 10 ± 4 min and operational time was 27 ± 8 min.

**Conclusions:**

We describe the creation of an ex vivo bovine kidney flow model for the preclinical evaluation of different embolic materials. The flow model can be modified to provide extensive bench testing and it is a promising tool for hands -on training in basic and advanced embolization techniques .

## Introduction

Therapeutic vascular embolization procedures represent established treatment options for a variety of conditions such as hemorrhage, tumor, and vascular malformations (Moreira and An 2003). Preclinical testing of embolic materials has been extensively described in live animals (Oh et al. 2015; Barbosa L de et al. 2009; Sommer et al. 2011; Siskin et al. 2003). It is essential to study and test novel embolic materials in animal tissue prior to human clinical trials; however formalized in-vivo trials may be taxing and resource consuming. Bench testing to characterize the behavior of novel embolic materials in an organ flow model may aid in selecting promising materials to then proceed into live animal studies. It may prove beneficial to establish a simple ex-vivo perfusion model for the pre-clinical evaluation of embolic agents.

To our knowledge, there is no description of an ex- vivo perfusion model of a bovine kidney to deploy embolic materials. The bovine kidney’s total volume and vascularity size facilitate the testing of embolic agents, without requiring specialized angiography catheters (Szymanski et al. 2018). Additionally, the arterial vasculature of the bovine kidney is similar to the human kidney including total number of primary segmental arteries (Szymanski et al. 2018). Unlike humans, the bovine renal artery splits before the hilum decreasing the technical difficulty of revealing the vessels with dissection of the renal hilar structures (Carvalho et al. 2009). Thus, we hypothesized that it would be possible to successfully test the deployment of commercially available embolic materials in a bovine kidney under fluoroscopy using standard angiography tools. We aimed to develop a flow model using a bovine kidney connected to a perfusion pump for the purpose of future testing of embolic agents in an inexpensive and easily reproducible model. The model could be also used as a hands-on training tool to teach the residents fellows the basics of embolization techniques.

## Methods

### Preparing the pump apparatus

A decommissioned perfusion roller pump (COBE Laboratories, Arvada, CO) was used. An acrylic basin to contain the kidney was prepared by drilling two holes into the side of the basin, to accommodate insertion of the arterial and venous tubing from the pump into the basin. Using a Y connector, the venous inflow tubing was divided into 2 segments: one for cannulation to the renal vein and one free end of inflow tubing for aspiration of water from the basin. A small puncture was made along the large outflow tubing to insert a 7Fr vascular sheath (Terumo, Somerset, NJ) to provide intra-vascular access.

### Kidney preparation/cannulation

Adult bovine kidneys were harvested within 4 h from a local slaughterhouse (Wiatrek Processing Plant, Poth, TX) and transported to our laboratory on ice and stored at − 20 °C until further use. A total of six kidneys were collected, five left and one right. Left kidneys were preferred, as it was easier to cannulate them given the longer renal veins. Kidneys were not frozen more than 1 week, and all were thawed 24 h prior to experiments. A 16Fr Foley catheter (McKesson, Grapevine, TX) was used to cannulate the renal vein and secured by inflating the Foley balloon followed by purse string suture and a zip tie around the outer wall of the vein. The renal artery was cannulated with a piece of 15Fr stiff tube and secured with a purse string suture and two zip ties.

### Initiating kidney perfusion

**Fig. 1:**
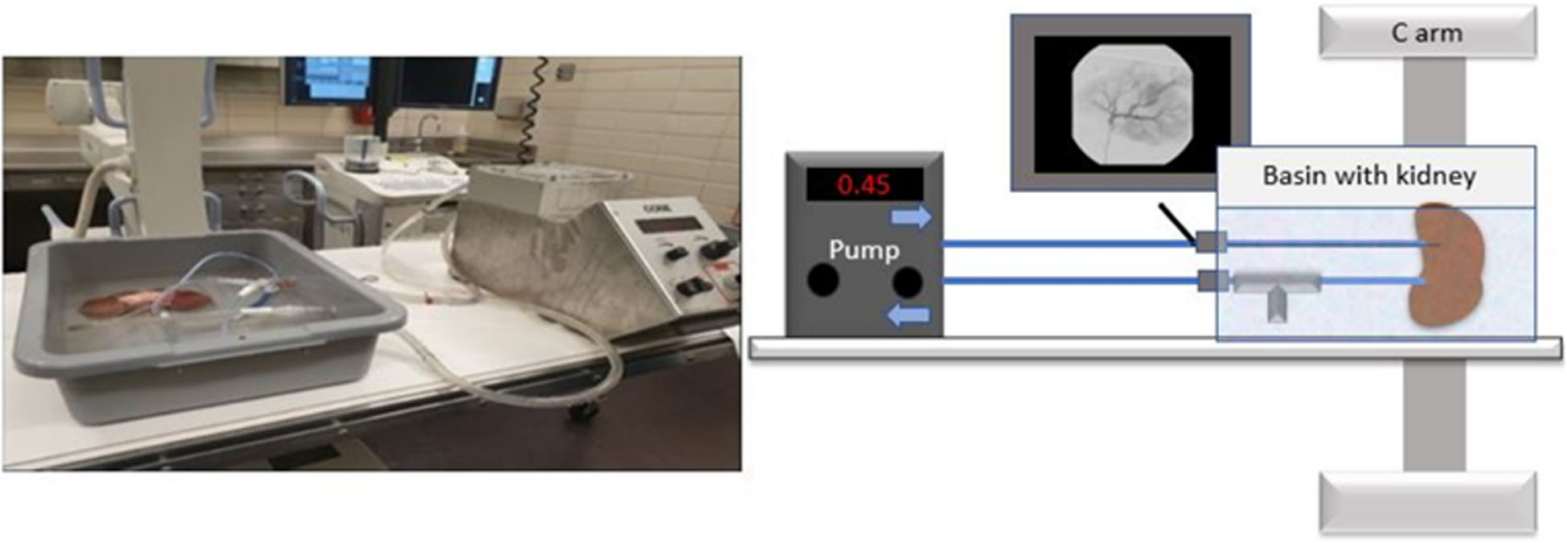
(Left) Photograph of the assembled flow model. (Right) Illustration of the perfusion system. The cannulated kidney is submerged in a basin. Tubing connects the cannulated kidney to the pump. Free end of Y connector on the venous tubing aspirates fluid for the pupmp. 5 Fr sheath is advanced into the arterial tubing for delivery of the selective catherization materials under fluoroscopy

The cannulated kidney was submerged in tap water inside the basin. The free end of the now cannulated Foley catheter was connected to the venous tubing with a Y-connector submerged in water. The basin was filled with 25 °C tap water to submerge the kidney and the free aspirating end of the Y connector. The system was purged of air by activating the pump at a flow rate of 0.40 L/min prior to attaching the arterial inflow connection to the renal artery. Once air bubbles were purged out of the tubing, the pump was deactivated. The proximal end of the 15Fr arterial cannula was connected to the arterial outflow tubing. Then, the pump was activated to a flow rate of approximately 0.49 L/min based on our pilot test. The assembled flow model is depicted in Fig. 1.

### Delivery of embolic materials

**Fig. 2:**
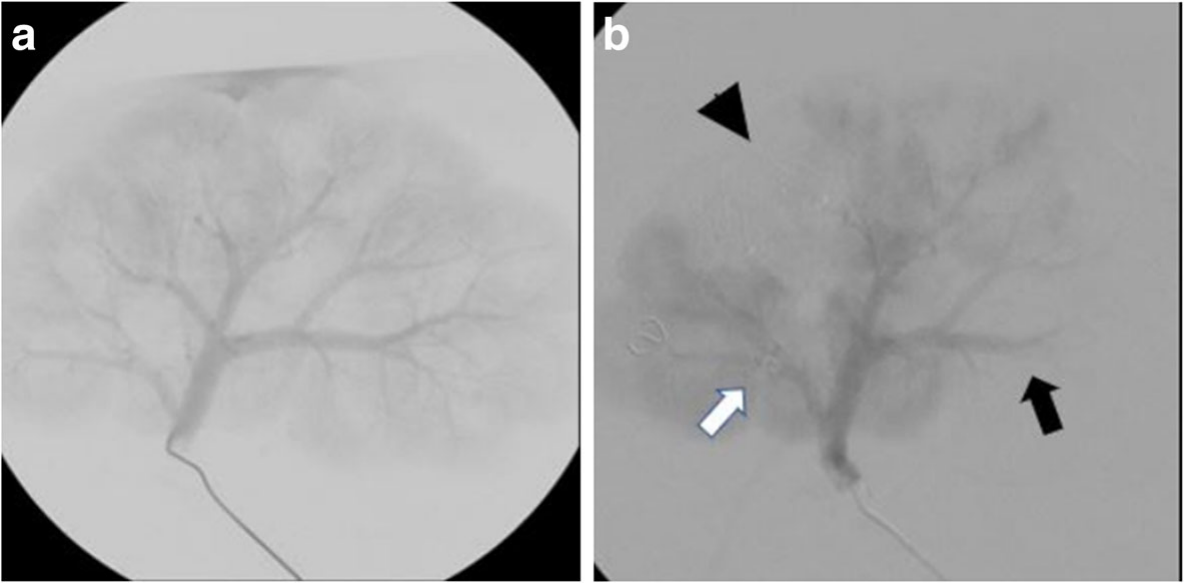
**a** Nonselective pre-embolization DSA run displaying renal arterial vasculature. **b** Nonselective post-embolization DSA run. Deployment of coil in cranial posterior segmental artery (white arrow) does not demonstrate appreciable embolic effect. Deployment of Gelfoam (black arrow) demonstrates decreased distal filling of the caudal posterior segmental artery. Glue (arrowhead) demonstrates complete embolization of the caudal secondary segmental artery

Angiography was conducted via a Siemens Arcadis C arm provided by our institution’s Department of Laboratory Animal Resources. This study involved the evaluation of three embolic materials: Tornado embolization coils (Cook Medical, Bloomington, IN), Gelfoam (Pfizer, New York, NY), and a glue mixture of Histoacryl (B Braun, Bethlehem, Pennsylvania) + Lipiodol (Guerbert, Princeton, NJ). Angiography and embolization for each kidney were attempted individually by a first-year diagnostic radiology resident and two medical students under the supervision of an interventional radiologist with 23 years of experience in vascular interventions. Each of the embolic materials were delivered to one of three segmental arterial branches in a single kidney (Fig. 2). A 5Fr cobra catheter (Boston Scientific, Marlborough, MA) with a Glidewire (Terumo, Somerset, NJ) was used for selection of segmental arteries and delivery of embolic materials**.** Once the catheter was placed in the renal artery, digital subtraction angiography (DSA) road mapping with Omnipaque contrast media (GE Healthcare, Wood Dale, IL) was conducted to outline arterial anatomy, assess for any intraparenchymal or extraparenchymal extravasation, and assist in selection of segmental branches for deploying the various embolic agents. For coil embolization, 3–4 mm 0,035-in. pushable coils were deployed using a Benston wire (Cook Medical, Bloomington, IN). Gelfoam particles were prepared by forcefully mixing a 2 cm X 2 cm piece of Gelfoam in a syringe filled with 5 mL of contrast media using a 3-way stopcock until a slurry was created. The Gelfoam slurry was slowly deployed with pulsatile boluses under fluoroscopy until stasis was observed. The volume of Gelfoam required to achieve stasis was recorded. For the deployment of glue, a mixture of 2 mL of lipiodol and 1 mL of Histoacryl was injected at a slow continuous rate until a cast of glue and subsequent stasis was visualized in the targeted segmental arteries. Glue infusion was preceded and succeeded with a 3 mL flush of the catheter with D5W.

### Data acquisition/analysis

Kidney weight upon delivery to the lab was recorded. Approximate freeze time was recorded to assess an effect on kidney tissue quality, feasibility of angiography, and arterial segmental selection. Cannulation time was collected to assess the reproducibility and difficulty of constructing the model. A pre-embolic DSA was performed to assess the variability in vascular anatomical architecture. The success rate of segmental artery selection was reported to determine whether it was feasible to execute at minimum three embolic experiments within individual segments of each single kidney. The success rate of embolic agent deployment was recorded to assess the model’s ability to serve as an embolic deployment mimic. Post-embolic nonselective DSA images were performed and interpreted to assess the embolic effect of the agents. Total operational time and fluoroscopy dose were recorded to assess the feasibility, practicality, and safety of the experiment. Additionally, the above data points were collected for a single right kidney for direct comparison to left kidney procedural outcomes.

## Results

The average weight of the six collected kidneys was 0.752 kg ± 0.094 kg. Five kidneys were cannulated with no complications and an average cannulation time of 21.6 min +/− 3.0 min. Kidney six, with a cannulation time of 46 min, was excluded as an outlier in this analysis due to multiple spontaneous decannulations of the arterial side. Kidneys were perfused with an average flow rate of 0.49 ± 0.07 L/min. Non-selective DSA demonstrated overall homogeneity in the vascular anatomy of bovine kidneys, with the renal artery dividing into a cranial and a caudal primary segmental arteries to supply the cranial and caudal poles, respectively. It was observed that typically the mid-zone of the kidney was supplied via a secondary segmental artery which came off the caudal primary segmental artery. In five kidneys, three distinct segmental arteries were identified and successfully individually selected via a 5Fr cobra catheter with Glidewire assist. All embolic materials were deployed as per Table 1. While the volume of glue mixture was fixed at approximately 3 mL, the average amount of Gelfoam required to achieve stasis was variable with an average deployment volume of 12 mL (+/− 3.5 mL). Post-op analysis revealed average dose area product to be 12.9 Gy.cm2 ± 1.8 Gy·cm2 and total fluoroscopy time to be 10 min ± 4 min, (*n* = 4). Overall operational time (time from first to last image) was 27 ± 8 min (Table 2).

**Table 1:** Characterization of Embolic Agent Targets by Renal Poles

	Cranial	Mid-Zone	Caudal	Cannulation Success (Cranial, Mid, Caudal)
1st Kidney	Coil	Glue	Gelfoam	+, +, +
2nd Kidney	Coil	Glue	Gelfoam	+, +, +
3rd Kidney	Gelfoam	Coil	Glue	+, +, +
4th Kidney	Gelfoam	Coil	Glue	-, +, +
5th Kidney	Gelfoam	Coil	Glue	+, +, +
6th Kidney	Gelfoam	Coil	Glue	+, +, +

+ = Successful Cannulation

- = Unsuccessful Cannulation

**Table 2:** Perfusion model data and specifications

	Perfusion rate (L/min)	Freeze time (weeks)	Cannulation time (min)	Operational time (hours)	Fluoroscopy time (sec)	Dose area product (Gy·cm2)
1st Kidney	0.41	2	24	30	Not collected	Not collected
2nd Kidney	0.48	3	17	20	Not collected	Not collected
3rd Kidney	0.50	0.5	23	21	351	13.5
4th kidney	0.60	0.5	24	18	425	15.2
5th kidney	0.50	0	20	38	795	11.5
6th kidney	0.43	4	46*	33	825	11.5

**Fig. 3:**
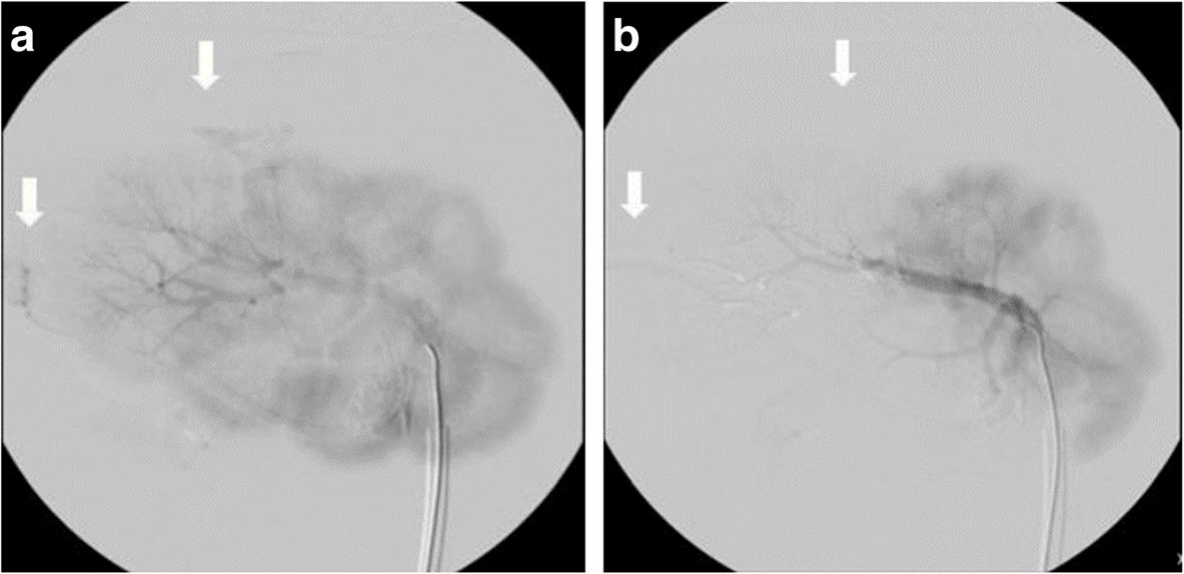
**a** Nonselective pre-embolization DSA displaying areas of contrast extravasation (white arrows). **b** Nonselective post-embolization DSA. Deployment of glue in the caudal posterior segmental artery (white arrows) demonstrates appreciable embolic effect evidenced by the decreased extravasation of contrast

As per post-embolic DSA runs, no stasis was appreciated with any of the metallic coils and a significant amount of Gelfoam was required to achieve stasis; complete stasis was observed in segments treated with glue (Fig. 2). Of note, upon pre-embolic DSA runs, contrast extravasation was present in the secondary segmental artery of kidney three and diffusely in kidneys five and six. Extravasation was not observed in branches after embolization (Fig. 3).

## Discussion

We developed an ex- vivo flow model using a bovine kidney for the purpose of future testing embolic agents in an inexpensive and easily reproducible model. The above results validate the model via DSA which clearly visualized the arterial anatomy and demonstrated a pulsatile flow with parenchymal blush and venous return on all the kidneys. Contrast extravasation was noted in some kidneys due varying degrees of parenchymal damage prior and upon perfusion that were related to tissue degradation of the frozen tissues, despite this limitation, we noticed that foci of contrast extravasation decreased after deployment of embolic materials, which implies that embolic agents were retained in the vascular bed of the selected artery, and that novel embolic materials could be preliminarily tested using a similar flow model to characterize embolic agent behavior and vascular distribution effects. In theory, the areas of contrast extravasation could also serve as teaching model to simulate bleeding control with embolization.

The preparation and operation of this flow model was consistently successful in the hands of a radiology resident and two medical students under the supervision of an experienced interventional radiologist. Thus, it can serve as an educational tool for radiation safety teaching, DSA, road mapping and selective angiography training, and practice techniques deploying embolic materials in a low-risk context. The bovine renal anatomy demonstrated easy identification and isolation of arterial vasculature supplying each kidney segment leading to precise application and testing of Gelfoam, glue, and embolization coils. Compared with animal models, advantages of this ex- vivo flow kidney model include lower associated costs, minimal structural requirements, and ease of preparation without the ethical concerns related to live animal use.

Notably, all six kidneys showed similar embolization efficacy results upon post-embolic DSA runs, particularly a diminishing degree of stasis as time passed after Gelfoam embolization. Under normal physiologic settings, Gelfoam promotes hemostasis by hastening the development of and providing structural support to thrombus formation (Kohda et al. 1986). Gelfoam has been successfully used in various clinical scenarios including hemorrhage secondary to trauma (Ben-Menachem et al. 1991), bone cancer (Feldman et al. 1975), renal cell carcinoma (Bracken et al. 1975), and uterine fibroid artery embolization (Siskin et al. 2000). The absence of an intact coagulation cascade in the perfusion model likely played a significant role in the deteriorating embolic effects of the Gelfoam. The lack a coagulation cascade also explains the failure of the coils in achieving stasis. This limitation must be taken into consideration when testing embolic materials that require specific physiological environments which are not present with ex -vivo models. The use of blood in this model may provide a better environment to test the effects of different embolization agents.

In this model, glue was the most effective embolic agent because it was not dependent on a coagulation cascade compared with Gelfoam and coils; however, utilization of glue demonstrated potential complications. In two kidneys glue adhered to the catheter tip during catheter removal post-deployment. In one kidney, the adhered glue dislodged into an unintended secondary segmental artery. These complications can also occur in the clinical practice. To prevent glue adhesion to the catheter and potential non-target embolization, the use of a microcatheter is recommended with a rapid removal from the glue cast .

Our study had several limitations. First, we used simple tap water as the perfusate. An isotonic buffer solution or blood may be more desirable to more closely mimic normal hydraulic physiology. Other perfusion models describe the use of heparinized saline or other more physiologic buffers to preserve tissue for transplantation and in ex- vivo radiofrequency ablation studies of the bovine liver (Orsi et al. 2011; Lubienski et al. 2006). Many organ perfusion models exist with protocols to extend ex-vivo viability for transplanting the kidney, liver, combined liver-kidney, lungs, heart, pancreas and small bowel using various buffers (Kumar et al. 2017; Tolstykh et al. 2002; Czogalla et al. 2016; Gao et al. 2020). We used tap water which may lead to changes in endothelial vasculature and renal parenchyma caused by a hypotonic solution. Secondly, kidneys were stored at -20C for variable periods of time before they were cannulated for the procedure. The extended time in a frozen state may have led to weakening of the renal parenchyma which was noted in some kidney models that had contrast extravasation. Ideally, the kidneys should be prepared and used for the experiment within the same day of procurement without frozen storage; however, harvesting kidneys on the same day of experiments is rarely feasible. Therefore, preservation techniques should be further explored as the model is refined. Finally, we did not have histological analysis of kidneys, pre and post embolization. Another limitation we encountered was the difficulty of maintaining the flow within the closed circuit without spontaneous decannulation of the arterial cannula. We later realized that our flow rates of 0.49 ± 0.07 L/min were too high. Slower, more physiologic flow rates to keep the systolic pressures between 60 and 80 mmHg would likely help prevent these issues in future models (5,7).

## Conclusions

We describe the successful creation of a simple pulsatile perfusion model of a bovine kidney obtained from a slaughtered animal for testing embolic agents in an inexpensive and easy way. Given the success of the perfusion model with clinically established agents, the model may present a valid means for pretesting novel embolic agents prior to proceeding to in vivo trials. Additionally, this model can serve as a realistic educational tool to teach basic angiographic techniques such as selective vessel catherization, use of road mapping and DSA, and basic and advanced embolization techniques to inexperienced trainees.

## Data Availability

Data is available to review.

## Abbreviations

DSA: Digital subtraction angiography
